# Skin-Grafting

**Published:** 1905-08-05

**Authors:** 


### SKIN-GRAFTING.

In the course of a very interesting paper on skin-grafting, Mr. A. Young[1] makes a strong plea for the use of grafts which are composed of the whole thickness of the skin. The grafts, which may be two, or three, or more inches in length, can be taken from a recently amputated limb, or from the patient himself. In the latter case the operation is carried out as follows: By two incisions extending to the deep fascia a long strip of skin is removed from some convenient situation, for example, the anterior part of the thigh. The skin thus removed is at once transferred to some warm sterile normal saline solution, while the wound in the thigh is sutured, dressed and bandaged. The strip of skin is now taken out of the saline solution and laid with the outer surface downwards in the palm of the operator's left hand, while with a pair of sharp scissors curved on the flat he quickly cuts off all adherent fat. When this has been done the surface about to be grafted is gently irrigated with warm saline solution and the grafts are applied. Mr. Young disapproves of scraping or rubbing the granulations previous to laying the grafts, on the grounds that the bleeding caused is a distinct drawback, and that the grafts will readily "take" when laid directly on intact healthy granulations. To retain the grafts in position a single layer of wide-meshed muslin is placed over them, and this is covered with several layers of sterile gauze moistened with sterile saline solution. A large sheet of gutta-percha tissue is placed over these dressings to retain the moisture, and over this a copious swathing of wool is bandaged lightly and equably. Subsequently, all the dressings except the wide-meshed muslin are removed daily, any discharge which has exuded is washed away by a gentle stream of saline solution, and a dressing similar to the first one is applied.

The chief advantage urged by Mr. Young in favour of this method is that the grafts almost never fail, while probably it will have been the experience of most surgeons who have frequently made use of Thiersch's operation to have met with many failures. Sometimes the grafts do not "take" at all, while quite commonly, after growing well for a time, they ulcerate, and show other evidences of deficient vitality. The objections to removing large strips of skin from other parts of the patient's own body in order to obtain the grafts is fairly obvious, and it is doubtful whether the cautious surgeon will resort to the method in ordinary cases unless he has already tried Thiersch's method and found it to fail.

[1] International Clinics, vol. i., 1905.

